# Integrative web-based analysis of omics data for study of drugs against SARS-CoV-2

**DOI:** 10.1038/s41598-021-89578-6

**Published:** 2021-05-24

**Authors:** ZhiGang Wang, YongQun He, Jing Huang, XiaoLin Yang

**Affiliations:** 1 Department of Biomedical Engineering, Institute of Basic Medical Sciences Chinese Academy of Medical Sciences, School of Basic Medicine Peking Union Medical College, Beijing, 100005 China; 2 Unit for Laboratory Animal Medicine, Department of Microbiology and Immunology, Center for Computational Medicine and Bioinformatics, University of Michigan Medical School, Ann Arbor, MI 48105 USA; 3 Department of Respiratory and Critical Care Medicine, Chongqing General Hospital, University of Chinese Academy of Sciences, Chongqing, 400014 China

**Keywords:** Computational biology and bioinformatics, Drug discovery

## Abstract

Research on drugs against SARS-CoV-2 (cause of COVID-19) has been one of the major world concerns at present. There have been abundant research data and findings in this field. The interference of drugs on gene expression in cell lines, drug-target, protein-virus receptor networks, and immune cell infiltration of the host may provide useful information for anti-SARS-CoV-2 drug research. To simplify the complex bioinformatics analysis and facilitate the evaluation of the latest research data, we developed OmiczViz (http://medcode.link/omicsviz), a web tool that has integrated drug-cell line interference data, virus-host protein–protein interactions, and drug-target interactions. To demonstrate the usages of OmiczViz, we analyzed the gene expression data from cell lines treated with chloroquine and ruxolitinib, the drug-target protein networks of 48 anti-coronavirus drugs and drugs bound with ACE2, and the profiles of immune cell infiltration between different COVID-19 patient groups. Our research shows that chloroquine had a regulatory role of the immune response in renal cell line but not in lung cell line. The anti-coronavirus drug-target network analysis suggested that antihistamine of promethaziney and dietary supplement of Zinc might be beneficial when used jointly with antiviral drugs. The immune infiltration analysis indicated that both the COVID-19 patients admitted to the ICU and the elderly with infection showed immune exhaustion status, yet with different molecular mechanisms. The interactive graphic interface of OmiczViz also makes it easier to analyze newly discovered and user-uploaded data, leading to an in-depth understanding of existing findings and an expansion of existing knowledge of SARS-CoV-2. Collectively, OmicsViz is web program that promotes the research on medical agents against SARS-CoV-2 and supports the evaluation of the latest research findings.

## Introduction

The number of confirmed infections with the severe acute respiratory syndrome coronavirus 2 (SARS-CoV-2) is still increasing. It is hence extremely urgent to study and develop antiviral drugs and vaccines. Significantly and luckily, bioinformatics methods can contribute a lot to providing key information in drug discovery and drug function evaluation.

SARS-CoV-2 is a novel coronavirus that infects patients through angiotensin-converting enzyme 2 (ACE2), which is the receptor protein located in cell membranes of the lung, leading to pneumonia reaction^1^. It also attacks other tissues such as the renal tissue^2^ and testicles^3^. It is important that the interference of drugs on the gene expression profiles in various cells, analysis of drug-target protein network and immune cell infiltration may provide a comprehensive understanding of crucial target proteins. For example, Pahm et al. established a deep learning model based on the Broad Institute Connectivity Map (CMAP) dataset, and applied the model for patient-specific drug repurposing for COVID-19^4^. Das et al. also carried out a screening of potential drugs by comparing CMAP dataset and transcriptome data of SARS-CoV-2 infected patients^5^. If a drug’s targeted host receptors interact with SARS-CoV-2, this drug could be a potential candidate for the infection. For example, using mass spectrum technology, a systematic analysis was carried out to study all proteins interacting with SARS-CoV-2 to screen possible targets for drug therapy^6^. It should be emphasized that the immune response can benefit virus clearing and body recovery. However, it may also result in a fatal pulmonary inflammatory reaction in case of over-reacted immune responses and the presence of a cytokine storm^7^.

A great number of new potential drugs have been reported recently, with the rapid accumulation of COVID-19 related data. Some evidence has shown that many of those drugs (e.g., nucleotide analogue remdesivir, HIV protease inhibitors lopinavir and ritonavir, broad-spectrum antiviral drugs arbidol and favipiravir, as well as antiviral phytochemicals) could decrease the morbidity and mortality of COVID-19 patients^8^. However, there is a significant disparity among the foci of the various research on COVID-19. The data were collected from different tissues, from patients of different ages and different levels of infection severity. So it would be helpful to build an integrated data platform to reuse the data and carry on more comprehensive and flexible data analysis. And such data analysis could provide more hints about COVID-19 target proteins and new drug discovery.

Some integrated COVID-19 data analysis platforms have been developed in the past year. Overmyer et al. compared the multi-omics characteristics of patients with SARS-CoV-2 infection of different severity groups. An interactive visualized platform was created to facilitate the study of these multi-omics data, aiming to elucidate disease pathophysiology and predict disease outcome^9^. The established platform provides PCA, linear regression, differential expression analysis, and cluster analysis by heatmap based on the author’s own data. Zhu et al. developed an online coronavirus genomic, proteomic, and evolutionary analysis platform^10^. Dannon Baker et al. integrated the available SARS-CoV-2 sequence data and published their results on the public Galaxy platform in order to promote the exchange of data between different laboratories^11^. Besides, some frequently used bioinformatics databases also cover valuable information about COVID-19 and support COVID-19 data analysis. For example, DrugBank website^12^ provides drug information in a tabular form, including drug description, chemical structure, indication, target proteins, adverse reactions, interactions, etc. L1000 database^13^ is available for users to compare the upregulated and downregulated gene lists with the gene expression profiles of drug-treated cell lines, which is useful for mining potential drugs. So a comprehensive data analyzing platform, combining COVID-19 related research data from diverse origins, with knowledge in different areas, would be beneficial for inspiring new discoveries.

In this study, we established the OmicsViz web program, an online interactive analysis system that can be used to study the interference of thousands of drugs on gene expression in different cell lines, and to carry out functional enrichment analysis on those expression changed genes. OmicsViz can also generate complex drug-target networks and further extend the networks based on protein–protein interactions (PPIs) to help identify the potential drugs. It can also be used to compare the immune cell infiltration status of samples based on the grouped transcriptome data. Based on the grouped RNA-seq transcriptome data, the OmicsViz can be used to determine the immune cell infiltration status.

In this paper, we introduce many features of OmicsViz and provide demonstrations on how OmicsViz supports COVID-19 research. Our demonstration results show that OmicsViz is an effective web tool for evaluating newly discovered drugs and target proteins, and comparing the immune infiltration based on RNA-Seq data uploaded by users and user-defined sample groups. Significantly, these results are of great importance for promoting drug development.

## Results

### Effect of drugs against SRAR-CoV-2 on gene expression profiles in lung and renal cell line cells using the Drug Spectrum module

**Figure 1:**
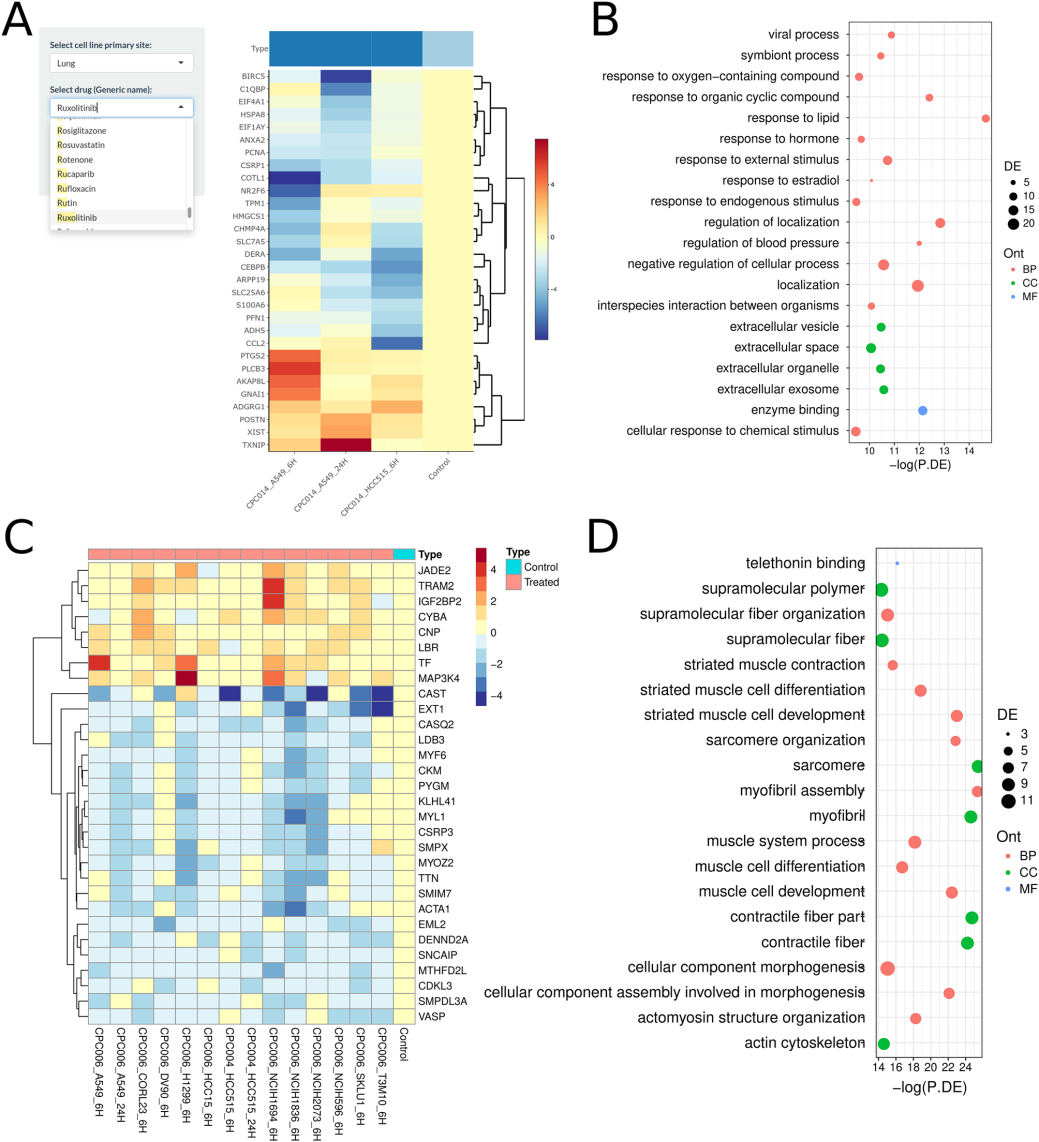
Effects of drugs on gene expression in cell lines. (**A**) Screenshot of ruxolitinib’s effect on the first 30 differentially expressed genes in lung cell line cells. The x-axis represents samples of lung cell lines (A549 and HCC515) treated with ruxolitinib for 6 and 24 h. Red, upregulated gene expression; Blue, downregulated gene expression. (**B**) The functional enrichment analysis of the 30 differentially expressed genes in (**A**). On the x-axis, − log(P.DE) is the negative logarithm with a base of 10 for *p*-value. The dot color represents three aspects of Gene Ontology. BP: Biological Process; MF: Molecular Function; CC: Cellular Component. The size of the dots indicates the number of differentially expressed genes in the corresponding functional annotation. The heatmap plot of the gene expression regulated by chloroquine in lung cell lines. (**D**) Functional enrichment analysis results of differentially expressed genes in (**C**).

The established web tool can be accessed by visiting http://medcode.link/omicsviz or http://hegroup.org:8080/omicsviz, and the *Drug Spectrum* module can be used to analyze the effect of any drug in LINCS 1000 database on the expressions of genes in various cell line cells. Besides, OmicsViz can generate interactive heatmaps and promote functional enrichment analysis of genes with altered expression. As of April 10, 2021, a total of 10,982 drug-cell line processing spectra had been generated. Utilizing this module, we analyzed the interference of ruxolitinib on lung cell lines and the effects of chloroquine on lung and renal cell lines. Ruxolitinib is a JAK inhibitor currently in Phase III clinical trials to treat COVID-19-induced cytokine storm^14^. Based on the first 30 differentially expressed genes selected in the ruxolitinib study, different gene expression profile changes were found in HCC515 and A549 cells, both derived from the lung (Fig. 1A). The Gene Ontology (GO) enrichment analysis of these genes revealed the enriched host responses to endogenous, external, and chemical stimuli (Fig. 1B), suggesting that the immune system’s active defenses against these stimuli in order to protect the organism and restore homeostasis^15^. For the chloroquine study with the lung cell lines, the enriched regulatory genes were primarily related to supramolecular fibers and muscle cell differentiation (Fig. 1C,D). While in the renal cell line cells, genes regulated by chloroquine exhibited an association with the regulation of immune response (Figure S1 A and B).

### Drug-target protein network analysis of coronavirus drugs

**Figure 2:**
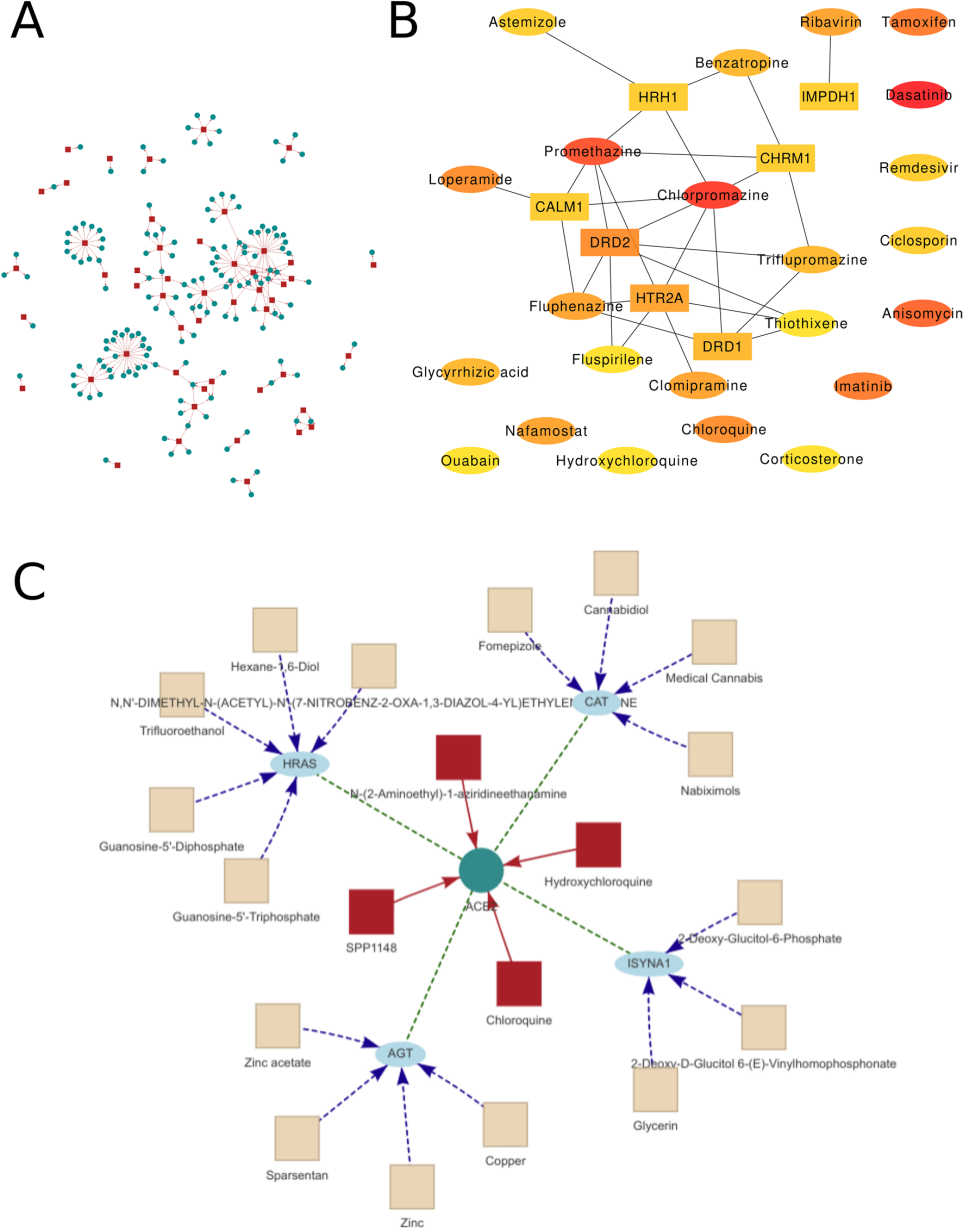
Drug-target protein network analysis for anti-coronavirus drugs. (**A**) The network analysis of 48 coronavirus drugs collected in our study. The red box represents the drug; The blue circle represents the target protein. The OmicsViz interactive interface lets you zoom in and out. The network can also be extended by combining data from PPIs such as the HINT database or STRING database. (**B**) The 30 hub nodes obtained by using the Maximum Clique Centrality method. (**C**) The extended network obtained by extending the ACE2 associated drug network with the HINT database. Peacock blue color node is ACE2. Red node indicates the drug targeted by ACE2 protein. Light blue node indicates the protein that interacts with ACE2 in the HINT database, and the light yellow node indicates the extended drug that targets light blue node.

As shown in Fig. 2A, the network formed by the obtained 48 drugs^16^ against coronaviruses such as SARS-CoV-2, SARS, and MERS from the public literature and their corresponding targets was analyzed using *Drug-target Network* module. The interactive view allows a specific area to be magnified to look at the nodes. Three options can be used to extend the network. To be specific, the ‘Extend drugs by shared targets’ means that when some drugs are input, their target proteins are extracted, and drugs targeting the same target proteins are displayed. The option of ‘Extend targets by HINT-db’ or ‘Extend targets by String-db’ indicated a possibility to add proteins that are known to interact with target proteins from HINT^17^ and STRING^18^ and extend new drugs at the same time. For the network formed by the studied 48 drugs and corresponding target proteins, the drugs involved in the analysis of their hub nodes included chlorpromazine, promethazine, fluphenazine, thiothixene, etc., and their target proteins were DRD2, HTR2A, CALM1, etc. (Fig. 2B). ACE2 is the spike protein receptor of SRA-Cov-2. Figure 2C shows the results of the analysis of the drugs targeting ACE2 and extended proteins by HINT, then extended drugs by drug-target interactions collected from DrugBank. Among these drugs, chloroquine and hydroxychloroquine target the ACE2 directly. Zinc is the extended drug targeting AGT, which interacts with ACE2. Nabiximols targets CAT, which interacts with ACE2 as well.

By incorporating the virus-host PPIs with drug-target interactions, the potential drugs can be further expanded in the *SARS-CoV-2 Network* module. This module provides the network of SARS-CoV-2 and its host PPIs based on the results of 481 interactions as obtained from a systematic study reported in *Nature*^6^. Furthermore, the virus-host PPIs can be significantly extended by adding 229 drug-target interactions (Figure S4A). In this extended network, fostamatinib, a tyrosine kinase inhibitor, targets 10 human proteins that interact with 6 viral proteins (i.e., M, Orf9b, N, Nsp9, Nsp12, and Nsp13) (Figure S4B). Fostamatinib inhibits MUC1 in the respiratory tract and has the potential to treat serious outcomes of COVID-19, including acute respiratory distress syndrome and acute lung injury^19^. Fostamatinib is currently under clinical trial as a treatment for COVID-19 (Clinicaltrials.gov ID: NCT04579393).

### Level of immune cell infiltration is associated with SARS-CoV-2 infection status and severity

**Figure 3:**
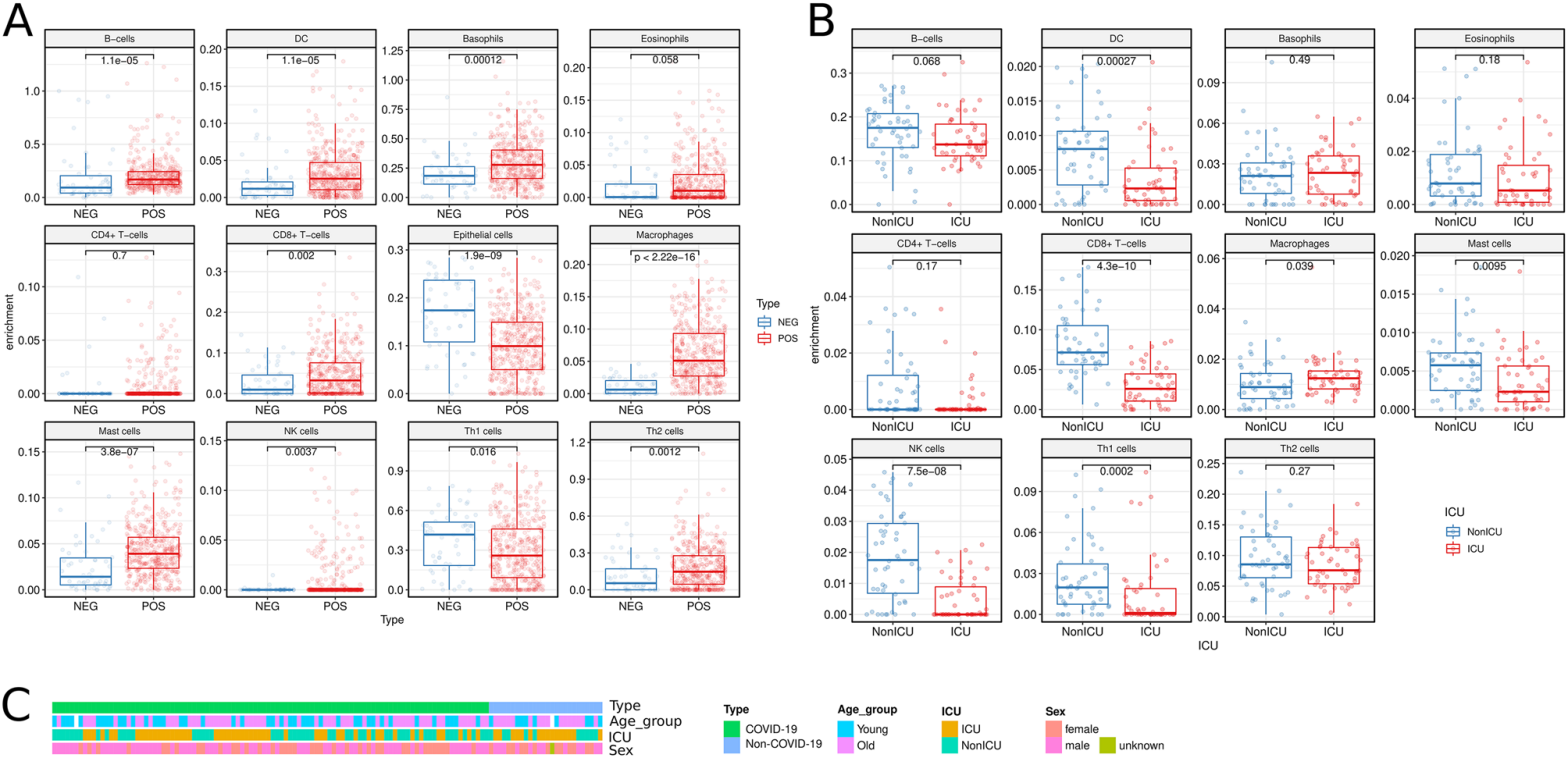
Comparison of immune cell infiltration based on RNA-seq data by using *Immune Infiltration* module. (**A**) Comparison of immune infiltration between SARS-CoV-2-positive and -negative samples in GSE152075 nasopharyngeal swabs. Most of the immune cells increased during infection. (**B**) The difference of immune infiltrating cells between the subjects entering and not entering ICU, using GSE157103 peripheral blood leukocyte samples. (**C**) The population characteristics of all subjects in the GSE157103 dataset. The age of samples was not significantly correlated with whether the samples were admitted to the ICU (chi-square test *p*-value = 0.087). However, compared to Figure S3 (Old vs. Young), the change in immune infiltration was in line with figure (**B**).

Inflammation reaction can be inferred from the cell composition of nasopharyngeal swab samples. RNA-seq data of nasopharyngeal swab samples (GSE152075) were obtained from the GEO database^20^. We compared the levels of immune cell infiltration between SARS-CoV-2-positive (n = 493) and -negative (n = 54) samples using gene signature-based methods. Corresponding results revealed that in response to SARS-CoV-2 infection, the numbers of many cell types, including B cells, dendritic cells (DCs), Basophils, CD8 + T cells, Macrophages, Mast cells, Natural killer (NK) cells, and Th2 cells, increased, while a decreasing trend was found in epithelial cells and Th1 cells (Fig. 3A).

Using peripheral blood leukocyte samples (GSE157103)^9^, we found that the immune cell changes in patients with SARS-CoV-2 infection were similar to those of nasopharyngeal swabs, as shown in Figure S2. Furthermore, we also compared the differences in immune cell infiltration between subjects > 60 years old and ≤ 60 years old, men and women, and different severity status groups. An obvious decrease was identified in the number of B cells, DCs, eosinophils, CD8 + T cells, Mast cells, NK cells, and Th1 cells in patients entering ICU, except macrophages (Fig. 3B). Meanwhile, although it is generally believed that the elderly occupies a higher proportion of ICU entries, there was no correlation between age and the status of entering ICU based on this dataset (Chi-square test *p*-value = 0.087, Fig. 3C). When different age groups were compared, the changes of immune cell infiltration in the elderly group were similar to those in the group of patients entering the ICU (Figure S3).

### Additional tools provided drug name mapping and RNA-Seq data conversion functions

**Figure 4:**
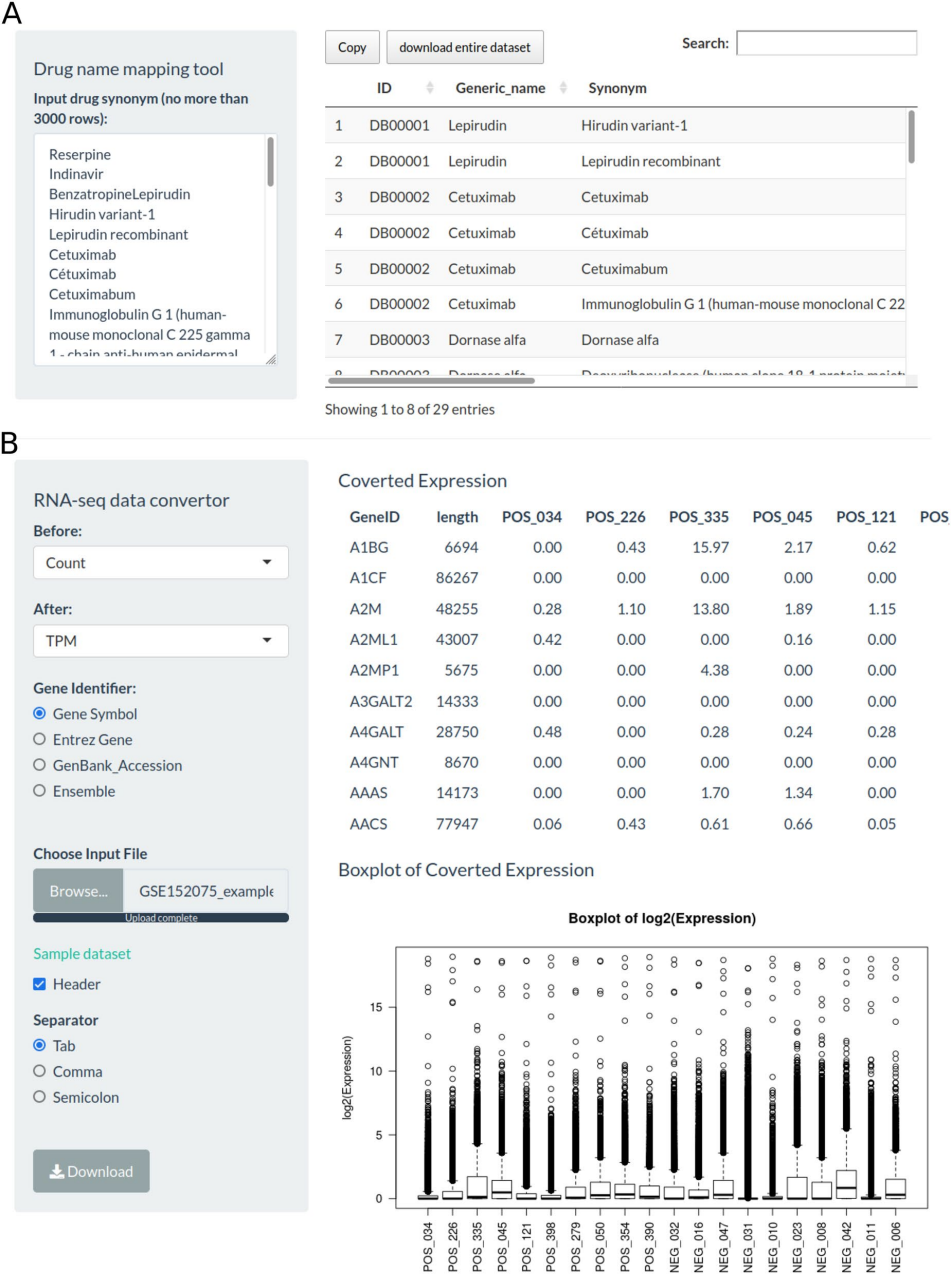
Screenshot of drug name mapping and RNA-seq measure conversion functions. (**A**) A total of 35,694 drug aliases of 13,339 drugs from the Drugbank database were included. You can enter or paste any drug names to find out their generic names. The obtained generic name can be used for drug-target protein network analysis. (**B**) Read counts data of RNA-seq data can be converted into TPM or FPKM data, and FPKM data can be converted into TPM data. Corresponding TPM data can be used to analyze immune infiltration.

In general, drugs in the literature may be named differently with different drug aliases, and the *Drug name mapping* function can be used to map the name of a drug to its generic name. It covered a total of 35,694 drug aliases for 13,339 drugs. The network analysis module of the web tool required the drug generic name for analysis. The different analyses needed different RNA-seq measures^21^, for example, TPM or FPKM data were required to compare the immune cell infiltration status with the xCell algorithm^22^. The gene expression data obtained from GEO and other public databases are usually represented by read counts, so *RNA-Seq data conversion* needs to be performed on those data for downstream immune infiltration analysis. (Fig. 4).

## Discussion

This paper introduces the features of our web program OmicsViz and demonstrates its usage for COVID-19 research. OmicsViz was used to examine the effects of chloroquine and ruxolitinib, two drugs actively tested for COVID-19 treatment, on lung and kidney cell line cells. Systematic analysis of the drug-target protein network of 48 anti-coronavirus drugs was performed with an analysis of the hub node. ACE2 bound drugs were extracted from drug-target interactions and extended by protein–protein interactions. The profiles of immune cell infiltration were compared between SARS-CoV-2-positive and -negative groups using pharyngeal swabs and peripheral blood leukocyte samples. Furthermore, the influences of age, gender, and severity on the outcome of immune cell infiltration of leukocytes were also examined. Significant results in our study were obtained with the support of OmicsViz.

Several interactive web tools focusing on real-time epidemic situations of the SARS-CoV-2 have been established. For instance, Dong et al. established a COVID-19 Dashboard, which can help to display the confirmed COVID-19 cases, deaths, and rehabilitation numbers in all affected countries/regions^23^. Similarly, COVID-NET, a population health monitoring system, was constructed to collect laboratory-confirmed data on hospitalization in 14 states of the United States^24^. There are other web tools that emphasize mining their own data. For example, Overmyer et al. generated large-scale multi-omics COVID-19 data^9^ and constructed a platform that can carry out principal component analysis, differential expression analysis, and cluster analysis, etc. In addition, Mehring et al. developed a web-based COVID-19 self-assessment tool providing guidance for patients with potential COVID-19 symptoms^25^.

However, most of these studies are limited to the analysis of the data obtained from their respective studies or focus on the pandemic and control of diseases from a macro perspective. There are only a few available platforms for the research of agents against COVID-19. Simultaneously, most platforms cannot carry out in-depth analyses on newly discovered drugs, target proteins, or user-updated data in the SARS-CoV-2 study. Besides, there is a lack of alternative platforms for medical or pharmaceutical researchers to simplify the data analysis process. In the cancer research area, web tools such as GEPIA2^26^, which incorporates The Cancer Genome Atlas (TCGA) and Genotype-Tissue Expression (GETx) data, have been widely used for biomarker discovery.

Our interactive OmicsViz web platform offers to the users the possibility to study the interference of arbitrary drugs on gene expression of various cell lines. It is available to evaluate the interaction network of one or more drug(s) or target protein(s) and to implement the extension by utilizing the common protein–protein interaction network database. The platform can be used to compare immune cell infiltration status based on RNA-Seq data. It also provided convenient tools for standardizing drug names and converting between different RNA-Seq measures. To our knowledge, OmicsViz is the first web tool to analyze multi-omics data for the COVID-19 study. Comparatively, PaintOmics is a general web resource for the pathway analysis and visualization of multi-omics data^27^. The strategies used in their work can be helpful in OmicsViz future development.

According to the results, chloroquine's interference with gene expression of lung cell lines is involved in differentiation of supramolecular fibers and muscle cells, but not antiviral response. While the regulated genes in renal cell lines exhibited primary association with immune response, which may exert an antiviral role. Wang et al. showed that both conventional drugs chloroquine and remdesivir showed a good inhibitory effect on COVID-19 in vitro^28^. Gautret et al. also believed a combination of hydroxychloroquine and azithromycin was effective in treating COVID-19^29^. Conversely, Tang et al. found no change in the time when SARS-CoV-2 RT-PCR tests were turned negative in COVID-19 patients treated with chloroquine/hydroxychloroquine^30^. Hoffmann et al. found that chloroquine was effective in preventing the invasion of SARS-CoV-2 in renal cell line cells, but not in lung cell line cells^31^. Our findings are consistent with the above findings, supporting that different cell lines have different responses to chloroquine and therefore to viruses.

Studies of ruxolitinib's effects on lung cell lines indicate that it mostly affects the response to endogenous and exogenous or chemical stimuli. Ruxolitinib has no antiviral activity but exerts an inhibitory role in cytokine storms by suppressing JAK kinase. Cao et al. found that COVID-19 patients treated with ruxolitinib had evidently reduced cytokine levels^32^, with no death cases reported as well, and the beneficial curative effect was also observed from CT images. It is suggested that ruxolitinib may have potential applications in reducing the mortality of patients with COVID-19 and reducing the sequelae.

The anti-coronavirus drug-target protein network analysis tool can be used to analyze ≥ 1 drug(s) or protein(s), and extend by PPIs database to benefit research of potential conventional drugs repurposing. In a recent study, it was documented that network-based coronavirus-host protein interaction analysis may be able to identify candidate SARS-CoV-2 drugs^33^. The method applied in our study shared similarity to their network-based approach for drug reuse. Significantly, the advantage in the current study resides in a more flexible platform that permits users to investigate newly discovered drugs and targets. As revealed in our study, the hub nodes in the network of 48 coronavirus drugs included chlorpromazine, promethazine, fluphenazine, thiothixene, and other drugs, while the target proteins included DRD2, HTR2A, CALM1, etc. These hub nodes may be the drugs or proteins that should be paid more attention to in the research and development of drugs against SARS-CoV-2. Among them, adrenaline and histamine antagonists have shown inhibitory effects on Ebola and Marburg viruses^34^. Both promethazine and fluphenazine are neurotransmitter inhibitors with activities against MERS-CoV and SARS-CoV^35^. In this regard, these drugs may be used in combination with antiviral drugs to treat COVID-19 patients. DRD2, one of the hub target proteins in our study, is a G protein-coupled receptor (GPCR), whose signal is mainly mediated by activated heterotrimeric GTP-binding protein (G protein). Studies have shown that GPCR can control signaling pathways that promote tumor cell survival via Akt^36^. Somanath’s research suggested that Akt can be used as a potential therapeutic target to treat patients with severe COVID-19^37^. Its inhibitors can also inhibit the expression of ACE2, suggesting that targeting Akt receptor in patients with COVID-19 may be a potential therapeutic strategy. In the extended network of drugs-ACE2 interactions by HINT PPIs, we revealed that the extended proteins included CAT, HRAS, AGT, and ISYNA1. These proteins may be potential drug targets, which was consistent with the findings of Xia et al.^38^. Their study disclosed that Zinc with AGT as its target protein can resist other virus infections, which was quite important for regulating immune function and reducing inflammation. Therefore, nutritional intervention could be an option for preventing SARS-CoV-2 and alleviating the progression of COVID-19^39^. Drug repositioning research using computational docking simulations is also very meaningful. For example, bioactive components from green tea and other herbs were tested against the SARS-CoV-2 virus by docking technology^40–42^. However, the docking process requires a heavy computing burden. So, the docking function is currently not integrated into OmicsViz.

The Immune infiltration module provides the function for comparison of the infiltration of immune cells using RNA-seq data. In our study, it was found that when infected with SARS-CoV-2, the numbers of B cells, DC cells, Basophils, CD8 + T cells, Macrophages, Mast cells, NK cells, and Th2 cells increased while the number of Th1 cells decreased. The changes of immune cells in blood leukocyte samples were consistent with those in swab samples except for DC and CD8 + T cells. Compared to the control samples, the number of immune cells in patients with COVID-19 increased, suggesting that there is a strong inflammatory response in COVID-19 patients^43^. Th2 cells can secrete IL-4, IL-5, IL-6, IL-9, IL-10, and IL-13, which have been shown to promote the proliferation and differentiation of B cells^44^. These results support our findings of more Th2 cells and fewer B cells. However, since epithelial cells are the main cells in swab samples and their proportion is easily influenced by collection operation, data correction is critical for the analysis of cell composition heterogeneity^45^. Despite the limitations, the measurement of the cells’ composition in nasopharyngeal swabs can provide useful information for assessing the infection and immunity of patients, and thus, assist in the treatment of COVID-19.

We also observed decreased levels of immune infiltrating cells in peripheral blood samples from ICU patients than those of non-ICU patients, suggesting that these patients were immune exhausted. The infiltration of immune cells in elderly patients was also decreased compared with young patients. As there was no statistical correlation between the age and the status of entering the ICU, we concluded that the disease mechanism of the elderly may be different from that of patients with severe disease. These results may suggest that anti-cytokine therapy can be used in the early stage of the disease to reduce immune system activation or exhaustion^46^.

In this paper, we present the application of OmicsViz for COVID-19 research. Function modules, such as the drug-cell interference spectrum analysis, the drug-target protein network analysis, and immune infiltration analysis, play an important role in drug development research. The users can submit their own data to conduct the analysis mentioned above. Hence Omicsviz is a general tool for assisting new drug development.

Our tool will be continually improved to expand current capabilities and solve potential limitations in the future. We plan to add more drug study modules, such as the evaluation of literature and clinical trial information based on text mining technology. For the SARS-CoV-2 study, the infection process and host immunity provide clues for drug discovery, therefore it is necessary to have modules for virus-host interaction analysis and data visualization. By adding these modules, the OmicsVis may lead to new directions in drug research. One of the limitations is the lack of comprehensive drug information integration, including drug indications, side effects, mechanisms of action, and clinical trials, all of which are essential for drug repurposing research. In our platform, we will integrate the relevant knowledge bases and provide new corresponding functions to help users gain insight into drugs. Currently, the platform can analyze RNA-seq data uploaded by users for immune cell infiltration comparison, but it cannot import public data directly from GEO and TCGA, which are of great value for tumor research. Research strategies on tumors, including precisely targeted therapy and immunotherapy evaluations, may be helpful in the COVID-19 study. Furthermore, in the data integration process, sample information is complicated and annotated at different levels, and the introduction of domain ontology and interoperable ontology is required. Many interoperable ontologies such as the Coronavirus Infectious Disease Ontology^47^ and Cell Line Ontology^48^, have been developed and are applicable in our study. We believe that the addition of more annotations derived from ontologies, such as the annotations of drug functional components and mechanisms of action, can further enhance the data integration and mining capability of OmicsViz, facilitating an efficient drug repurposing process.

## Methods

For LINCS 1000 database^13^, the involved cells were classified according to the primary site of the source, including 20 cell lines (i.e., adipose, autonomic ganglia, blood, bone, breast, central nervous system, endometrium, hematopoietic and lymphoid tissue, kidney, kidney, large intestine, liver, lung, muscle, ovary, pancreas, prostate, skin, stomach, vascular system, and ESC).

A total of 19,811 small-molecule compounds were included in our analysis of which 1460 are in DrugBank and can be analyzed on our web tool^12^. Note that not all cell lines were treated with all the drugs. The data only with 6 h and 24 h of treatment were selected for analysis, and finally, there were 10,982 drug-cell lines treatment profiles.

Our study used Level 5 data, which is z-scoring standardization of the differential expression values by considering each experiment’s replicated samples. An up-regulated gene has expression > 0 while a down-regulated gene has expression < 0. Additionally, a virtual control sample was introduced to demonstrate the differential expression preferably, whose gene expression value was set to 0 in heatmap output. The heatmaply^49^ R package was used to visualize data.

To identify differentially expressed genes for samples more than 3, a moderated contrast t-test in limma package^50^ was used to select the top n items according to the order of the *p*-value. While for those with the sample size of < 3, the top n/2 genes with upregulated and downregulated differential expression were selected by Fold-change based on gene expression. For the differentially expressed genes identified above, the functional enrichment analysis was performed by the GO enrichment using the topGO function of the limma package. This function performs gene set over-representation analyses based on hypergeometric tests for GO terms. We used genes of human species as background genes and unadjusted *p*-value smaller than 0.05 was set to filter the enriched terms. If the number exceeds 30, only the top 30 terms with the smallest *p* values are rendered in the graph output. Using the above functions, we examined the interference of ruxolitinib on lung cell lines and the effects of chloroquine on lung and kidney cell lines.

A total of 17,504 drug-target interactions were downloaded from the DrugBank database (version 5.1.7, 2020-07-02)^12^, of which 7265 drugs and 3972 proteins. The interactions between the SARS-CoV-2 virus proteins and host proteins derived from the study on *Nature* journal^6^. The drug-drug interaction dataset, which was derived from the Twosides database (http://tatonettilab.org/offsides/), was also added to the OmicsViz server. The extension of PPIs network was achieved by using STRING^18^ and HINT^17^. For STRING (https://stringdb-static.org/download/protein.links.v11.0/9606.protein.links.v11.0.txt.gz), part of the human data was chosen to screen high-trust interactions with a score > 700, with a total of 839,224 interactions obtained finally. A total of 199,675 interactions were integrated from binary interaction and co-complex from the HINT dataset (http://hint.yulab.org/download/). It should be noted that when slightly more nodes were used as input, a very large network may be formed. Under this condition, the program will only present the original network and some results of 300 random selections from newly introduced interactions.

The hub node analysis of the network was carried out using the Maximal Clique Centrality method provided by Cytohubba^51^ plugin of Cytoscape^52^. A network analysis was conducted on the 48 drugs^16^ against coronaviruses such as SARS-CoV-2, SARS, and MERS, which were retrieved from the literature.

Inflammation may alter the cell composition of nasopharyngeal swabs and blood leukocyte samples. The xCell algorithm^22^, a gene signatures-based method learned from thousands of pure cell types from various sources, was applied primarily for cell heterogeneity comparison among samples. Following the determination of cell types by the user based on the biological knowledge, immune infiltration can be analyzed. The GSE152075^20^ data covered RNA-sequencing profiles of nasopharyngeal swabs from 430 individuals with SARS-CoV-2 and 54 negative controls. Since the clinical information of the samples is not available, we only compared the immune infiltration of 493 positive and 54 negative samples of SARS-CoV-2 using the Immune Infiltration module. Meanwhile, GSE157103 data was also collected from the GEO database^9^. Further study was conducted on the 102 SARS-CoV-2 infected samples with different degrees of severity. Also, comparisons on immune cell infiltration were carried out between subjects entering the ICU and not, and between subjects aged > 60 and ≤ 60 years old, and between men and women.

The drugs and their aliases were obtained from DrugBank^12^ database which is in xml format. We borrowed the conversion from Lior Pachter^53^ for the conversion between TPM and FPKM. Meanwhile, the development of the RNA-seq measures conversion module referred to the work of Sang Cheol et al.^21^.

## Supplementary Information

Supplementary Legends.

Supplementary Figure 1.

Supplementary Figure 2.

Supplementary Figure 3.

Supplementary Figure 4.
