# Supplementary material for: Integrative web-based analysis of omics data for study of drugs against SARS-CoV-2
Source: Sci Rep. 2021 May 24;11:10763. doi: 10.1038/s41598-021-89578-6 (PMC8144609; doi:10.1038/s41598-021-89578-6)
Supplement: Supplementary file 1 — Supplementary Legends. [file 41598_2021_89578_MOESM1_ESM.docx]

Figure S1. (A) The heatmap of gene expression regulated by chloroquine in renal cell lines. (B) Results of functional enrichment analysis of differentially expressed genes in A.

Figure S2. Comparison of immune infiltration profiles between infected and non-infected subjects using GSE157103 dataset. On the x-axis, C indicates coronavirus infection and NC means negative.

Figure S3. Comparison of immune infiltration profiles between subjects >60 and <=60 years of age using GSE157103 dataset.

Figure S4. (A) Extended network of virus-host PPIs by drug-target interactions. (B) A closer look at the drug fostamatinib, which targets 10 human proteins that interact with viruses.
