# Supplementary material for: Integrative web-based analysis of omics data for study of drugs against SARS-CoV-2
Source: Sci Rep. 2021 May 24;11:10763. doi: 10.1038/s41598-021-89578-6 (PMC8144609; doi:10.1038/s41598-021-89578-6)
Supplement: Supplementary file 2 — Supplementary Figure 1. [file 41598_2021_89578_MOESM2_ESM.pdf]

Integrative Web-based Analysis of Omics Data for Study of Drugs Against SARS-CoV-2

ZhiGang Wang[1], YongQun He[2], Jing Huang[3], XiaoLin Yang[1*]

[1] Department of Biomedical Engineering, Institute of Basic Medical Sciences Chinese Academy of Medical Sciences, School of Basic Medicine Peking Union Medical College, Beijing, 100005, China.

[2] Unit for Laboratory Animal Medicine, Department of Microbiology and Immunology, Center for Computational Medicine and Bioinformatics, University of Michigan Medical School, Ann Arbor, MI 48105, USA.

[3] Department of Respiratory and Critical Care Medicine, Chongqing General Hospital, University of Chinese Academy of Sciences, Chongqing, 400014, China

* Correspondence should be addressed to X.Y. (email: yangxl@pumc.edu.cn)

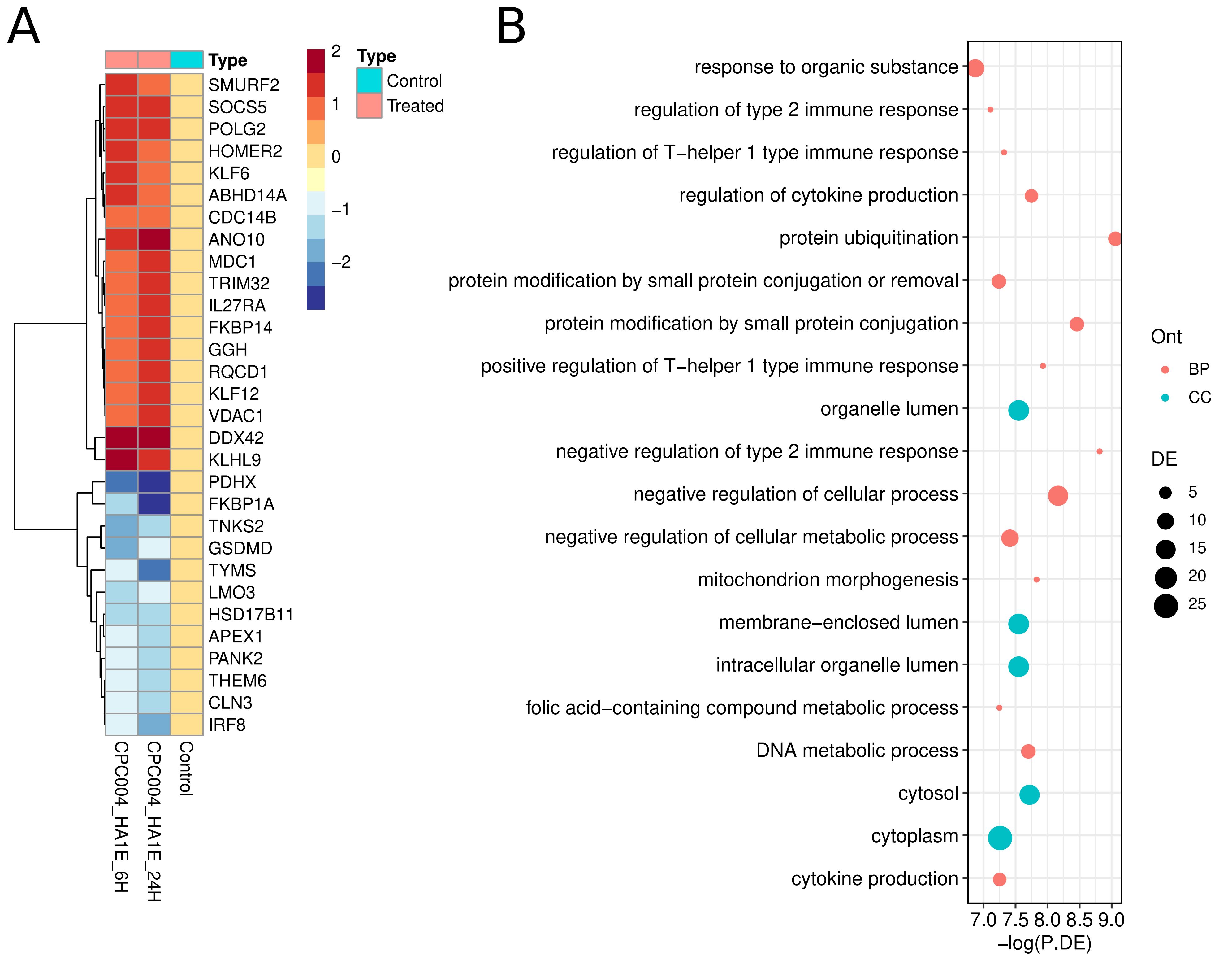

A
B
Type
SMURF2
SOCS5
POLG2
HOMER2
KLF6
ABHD14A
CDC14B
ANO10
MDC1
TRIM32
IL27RA
FKBP14
GGH
RQCD1
KLF12
VDAC1
DDX42
KLHL9
PDHX
FKBP1A
TNKS2
GSDMD
TYMS
LMO3
HSD17B11
APEX1
PANK2
THEM6
CLN3
IRF8
CPC004_HA1E_6H
CPC004_HA1E_24H
Control
2
1
0
−1
−2
Type
Control
Treated
response to organic substance
regulation of type 2 immune response
regulation of T−helper 1 type immune response
regulation of cytokine production
protein ubiquitination
protein modification by small protein conjugation or removal
protein modification by small protein conjugation
positive regulation of T−helper 1 type immune response
organelle lumen
negative regulation of type 2 immune response
negative regulation of cellular process
negative regulation of cellular metabolic process
mitochondrion morphogenesis
membrane−enclosed lumen
intracellular organelle lumen
folic acid−containing compound metabolic process
DNA metabolic process
cytosol
cytoplasm
cytokine production
7.0 7.5 8.0 8.5 9.0
−log(P.DE)
Ont
BP
CC
DE
5
10
15
20
25
