# Supplementary material for: Integrative web-based analysis of omics data for study of drugs against SARS-CoV-2
Source: Sci Rep. 2021 May 24;11:10763. doi: 10.1038/s41598-021-89578-6 (PMC8144609; doi:10.1038/s41598-021-89578-6)
Supplement: Supplementary file 4 — Supplementary Figure 3. [file 41598_2021_89578_MOESM4_ESM.pdf]

Integrative Web-based Analysis of Omics Data for Study of Drugs Against SARS-CoV-2

ZhiGang Wang[1], YongQun He[2], Jing Huang[3], XiaoLin Yang[1*]

[1] Department of Biomedical Engineering, Institute of Basic Medical Sciences Chinese Academy of Medical Sciences, School of Basic Medicine Peking Union Medical College, Beijing, 100005, China.

[2] Unit for Laboratory Animal Medicine, Department of Microbiology and Immunology, Center for Computational Medicine and Bioinformatics, University of Michigan Medical School, Ann Arbor, MI 48105, USA.

[3] Department of Respiratory and Critical Care Medicine, Chongqing General Hospital, University of Chinese Academy of Sciences, Chongqing, 400014, China

* Correspondence should be addressed to X.Y. (email: yangxl@pumc.edu.cn)

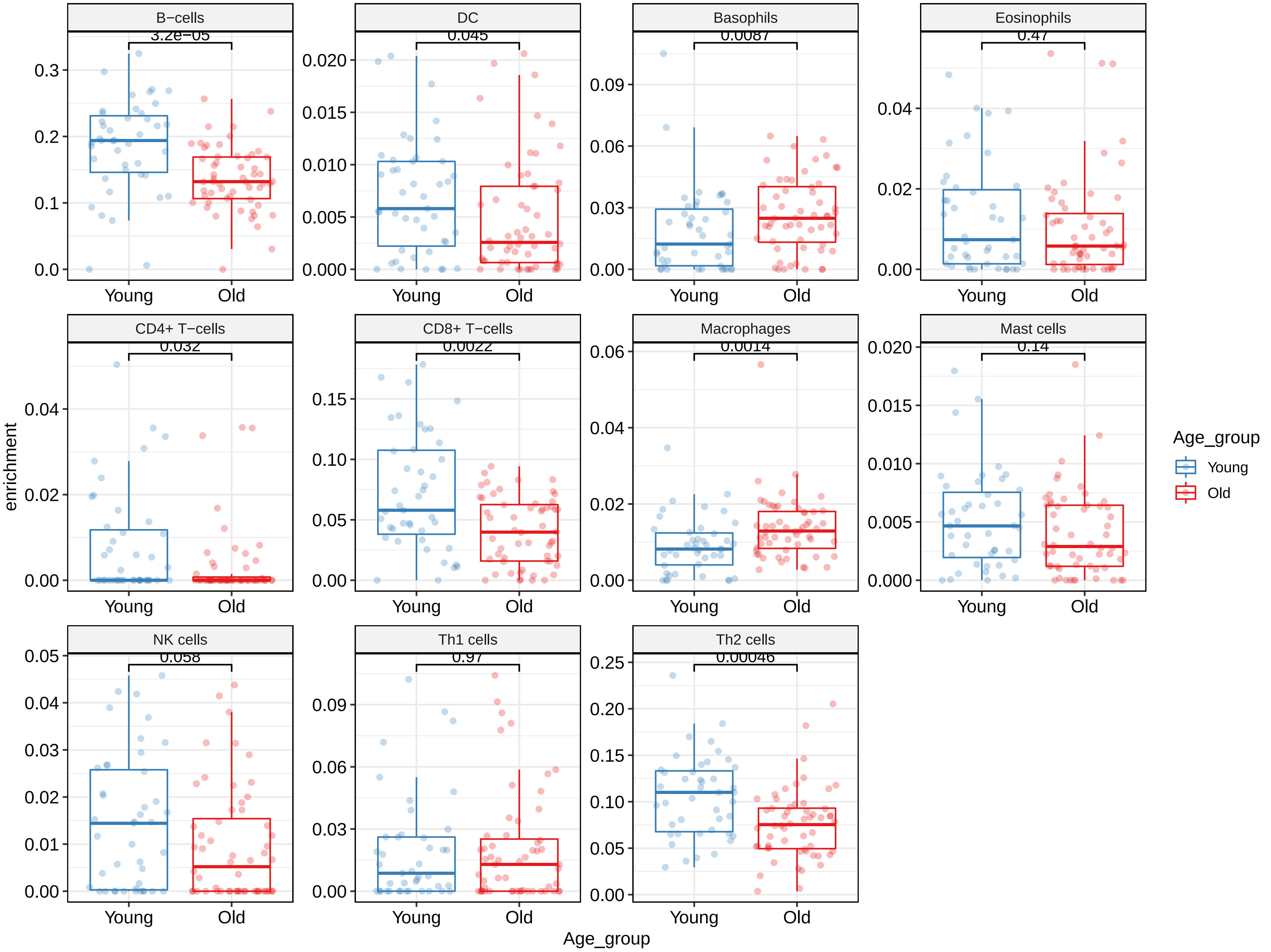
B−cells
3.2e−05
DC
0.045
Basophils
0.0087
Eosinophils
0.47
CD4+ T−cells
0.032
CD8+ T−cells
0.0022
Macrophages
0.0014
Mast cells
0.14
NK cells
0.058
Th1 cells
0.97
Th2 cells
0.00046
Young
Old
enrichment
Age_group
Age_group
Young
Old
