# Supplementary figures and images for: Integrative web-based analysis of omics data for study of drugs against SARS-CoV-2
Source: Sci Rep. 2021 May 24;11:10763. doi: 10.1038/s41598-021-89578-6 (PMC8144609; doi:10.1038/s41598-021-89578-6)

A

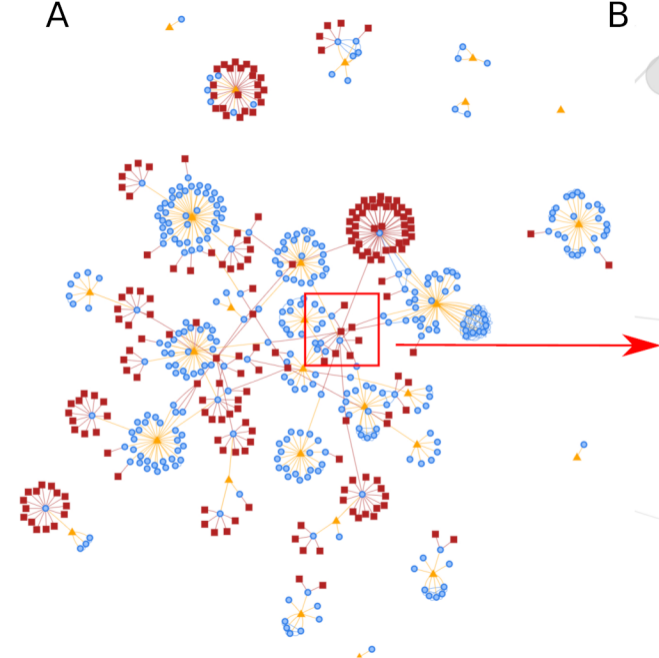

B

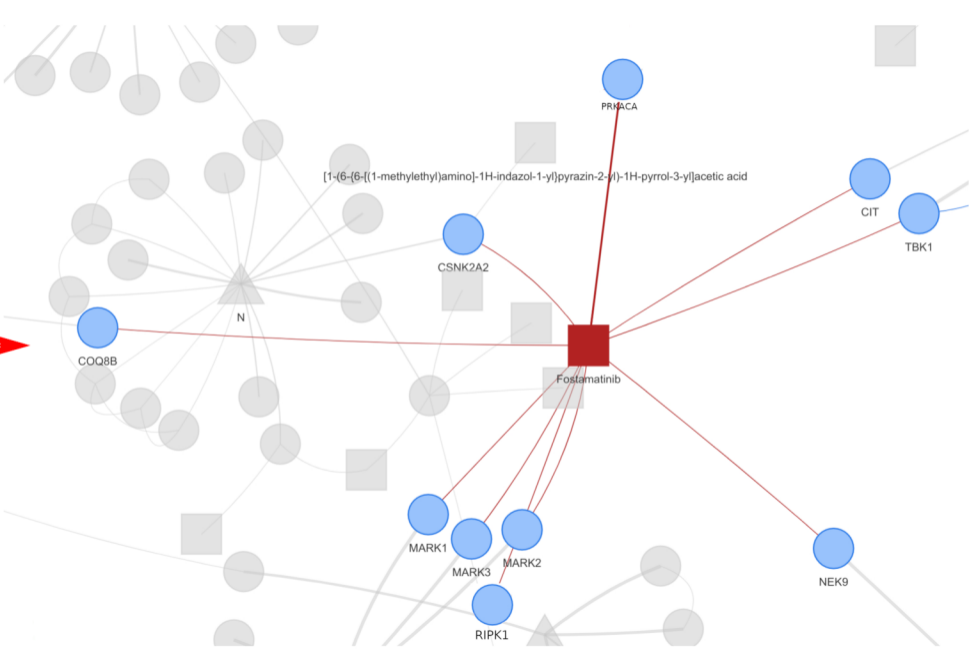

Supplement: Supplementary file 5 — Supplementary Figure 4. [file 41598_2021_89578_MOESM5_ESM.pdf]
